# Hesperetin Alleviates the Inhibitory Effects of High Glucose on the Osteoblastic Differentiation of Periodontal Ligament Stem Cells

**DOI:** 10.1371/journal.pone.0067504

**Published:** 2013-06-28

**Authors:** So Yeon Kim, Jin-Yong Lee, Yong-Duk Park, Kyung Lhi Kang, Jeong-Chae Lee, Jung Sun Heo

**Affiliations:** 1 Department of Maxillofacial Biomedical Engineering and Institute of Oral Biology, School of Dentistry, Kyung Hee University, Dongdaemun-gu, Seoul, South Korea; 2 Department of Preventive and Social Dentistry, School of Dentistry, Kyung Hee University, Dongdaemun-gu, Seoul, South Korea; 3 Department of Periodontology, School of Dentistry, Kyung Hee University, Dongdaemun-gu, Seoul, South Korea; 4 Institute of Oral Biosciences and School of Dentistry, Research Center of Bioactive Materials, Chonbuk National University, Jeonju, South Korea; Rush University Medical Center, United States of America

## Abstract

Hesperetin (3′,5,7-trihydroxy-4-methoxyflavanone) is a metabolite of hesperidin (hesperetin-7-O-rutinoside), which belongs to the flavanone subgroup and is found mainly in citrus fruits. Hesperetin has been reported to be an effective osteoinductive compound in various in vivo and in vitro models. However, how hesperetin effects osteogenic differentiation is not fully understood. In this study, we investigated the capacity of hesperetin to stimulate the osteogenic differentiation of periodontal ligament stem cells (PDLSCs) and to relieve the anti-osteogenic effect of high glucose. Osteogenesis of PDLSCs was assessed by measurement of alkaline phosphatase (ALP) activity, and evaluation of the mRNA expression of ALP, runt-related gene 2 (Runx2), osterix (OSX), and FRA1 as osteogenic transcription factors, as well as assessment of protein expression of osteopontin (OPN) and collagen type IA (COLIA). When PDLSCs were exposed to a high concentration (30 mM) of glucose, osteogenic activity decreased compared to control cells. Hesperetin significantly increased ALP activity at doses of 1, 10, and 100 µM. Pretreatment of cells with hesperetin alleviated the high-glucose-induced suppression of the osteogenic activity of PDLSCs. Hesperetin scavenged intracellular reactive oxygen species (ROS) produced under high glucose condition. Furthermore, hesperetin increased the activity of the PI3K/Akt and β-catenin pathways. Consistent with this, blockage of Akt or β-catenin diminished the protective effect of hesperetin against high glucose-inhibited osteogenic differentiation. Collectively, our results suggest that hesperetin alleviates the high glucose-mediated suppression of osteogenic differentiation in PDLSCs by regulating ROS levels and the PI3K/Akt and β-catenin signaling pathways.

## Introduction

Multipotent postnatal stem cells have been identified in oral tissues, such as those from dental pulp [1], exfoliated deciduous teeth [2], and periodontal ligaments (periodontal ligament stem cells [PDLSCs]) [3]. These dental stem cells are plentiful and easy to collect from tissue, and have been shown to be able to differentiate into bone, dental tissue, cartilage, and even neural tissue [4]–[6]. They are being studied for a number of disorders of connective tissues or neural tissues in the body including type I diabetes, damaged tooth structures, skeletal bone loss, and neurodegenerative diseases [7]–[9]. In particular, PDLSCs are capable of differentiating into an osteoblastic lineage, and are therefore considered a good regenerative medicine candidate for treatment of bone defects [10]. However, the biology of PDLSCs under various oral conditions needs to be clarified more precisely, because the condition of periodontal ligament structures is closely associated with risk factors including smoking, diabetes, and other infections [11], [12]. Among these pathological states, high glucose levels in diabetic patients may alter the regenerative capacity of PDLSCs. In this study, we investigated PDLSC responses to diabetic conditions and the effects of a natural compound, hesperetin, on these PDLSC responses to high glucose.

Flavonoids, which are naturally occurring polyphenolic compounds that form part of the human diet, have been reported to affect bone metabolism [13]. Among flavonoids, hesperetin (3′,5,7-trihydroxy-4-methoxyflavanone), which is a member of the flavanone subclass of flavonoids, is found mainly in citrus fruit. This flavonoid exists in nature in its glycoside form, hesperidin [14]. Hesperetin and its metabolites have several biological activities; they display antioxidant, anti-inflammatory, and lipid lowering effects [15], [16]. However, despite the accessibility of hesperetin, few studies have investigated whether hesperetin influences bone strength and osteoblast differentiation [17], [18]. Moreover, the mechanisms underlying the osteogenic effect of hesperetin are not fully understood. It has been reported that flavonoids and their metabolites regulate the phosphoinositide 3-kinase (PI3K), Akt, mitogen activated protein kinase (MAP kinase), and Wnt/β-catenin signaling pathways [19], [20]. Inhibition or stimulation of these pathways can modulate cellular functions including bone formation and regeneration processes by altering the expression of target molecules and genes in response to various microenvironmental conditions.

Hesperetin may be a potential candidate for promoting bone regeneration given its therapeutic efficiency and low cost.

In the present study, cells were cultured in medium supplemented with physiological (NG, 5.5 mM) or diabetic (HG, 30 mM) glucose levels to determine whether the high glucose level inhibited the differentiation of PDLSCs into an osteogenic lineage and to characterize the impact of hesperetin on this process.

## Materials and Methods

### Materials

Fetal bovine serum (FBS) was purchased from Gibco-BRL (Gaithersburg, MD, USA). Nicotine, α-bungarotoxin, and mecamylamine were obtained from Sigma Chemical Company (St. Louis, MO, USA). Collagen type I, osteopontin, Runx2, osterix, β-catenin, β-actin, goat-anti mouse, and goat-anti rabbit antibodies were supplied by Santa Cruz Biotechnology (Delaware, CA, USA). Unless otherwise specified, chemicals and laboratory-ware were purchased from Sigma Chemical Company and Falcon Labware (Becton-Dickinson, Franklin Lakes, NJ, USA), respectively.

### Periodontal Ligament Stem Cell Culture

Periodontal ligaments were obtained from extracted human molars, which were donated by the Department of Oral and Maxillofacial Surgery of Kyung Hee University. All subjects involved in this study were informed about the purpose and procedures of this study, which was approved by the Review Board of Kyung Hee University. Written informed consent was obtained from all donors and guardians on behalf of minor participants. Periodontal ligaments, collected from the middle third of the root, were cultured in α minimal essential medium (α-MEM) (Invitrogen, Carlsbad, CA, USA) containing 10% FBS, penicillin (100 U/mL) and streptomycin (100 µg/mL) (SIGMA, St. Louis, MO, USA) according to a previously described method [3]. After two passages, cells were subjected to magnetic isolation with antibodies to detect STRO-1 (mesenchymal stem cell marker) antigen (Millipore, Billerica, MA, USA) and magnetic beads (MiltenyiBiotec, Germany). The resulting STRO-1(+) cell population was cultured in α-MEM plus 10% FBS at 37°C in a humidified gas mixture of 5% CO_2_/95% air. All experiments were carried out with passage 4–7 cells. To assess the colony-forming efficiency, cells at passage 1 were seeded onto a 35 mm dishes. After 5 days of culture, cultures were fixed with 4% formalin, and then stained with 0.5% crystal violet (Sigma–Aldrich Co.) (Figure S1).

### Osteogenic Differentiation of PDLSCs

Differentiation was initiated by a switch to the osteogenic medium of α-MEM containing 5% FBS, 50 µg/mL ascorbic acid, 1 µM dexamethasone, and 3 mM β-glycerophosphate. In each experiment, cells were incubated in medium supplemented with 5.5 mM D-glucose (NG as a control), 30 mM D-glucose (HG), hesperetin at different concentrations (0.1, 1, 10, and 100 µM), or HG plus hesperetin for a designated number of days. The medium was changed every other day. Hesperetin was dissolved in dimethyl sulfoxide (DMSO) immediately before use, and the final concentration of DMSO did not exceed 0.1% (v/v) in any of the experiments. DMSO at 0.1% was used as a control.

### Alkaline Phosphatase Activity

Cells were washed twice with PBS and lysed in 50 mM Tris–HCl buffer (pH 7.0) containing 1% (v/v) Triton X-100 and 1 mM PMSF. Total protein was then quantified using the Bradford procedure [21]. The entire cell lysate was assayed by adding 200 µl of *p*-nitrophenylphosphate (pNPP) as a substrate (Sigma, USA) for 30 min at 37°C. The reaction was stopped by adding 3 N NaOH and the absorbance was read spectrophotometrically at 405 nm. Enzyme activity was expressed as mM/100 µg of protein.

### RNA Isolation and Real-time RT-PCR

Total RNA was extracted from cells using TRIzol reagent (Invitrogen, Carlsbad, CA), following the manufacturer’s instructions. Real-time quantification of RNA targets was then performed using a Rotor-Gene 2000 real-time thermal cycling system (Corbett Research, NSW, Australia) and a QuantiTect SYBR Green RT-PCR kit (QIAGEN, CA, USA). The reaction mixture (20 µl) contained 200 ng of total RNA, 0.5 µM of each primer, and appropriate amounts of enzymes and fluorescent dyes as recommended by the supplier. The Rotor-Gene 2000 cycler was programmed as follows: 30 min at 50°C for reverse transcription; 15 min at 95°C for DNA polymerase activation; 15 sec at 95°C for denaturation; and 45 cycles of 15 sec at 94°C, 30 sec at 55°C, and 30 sec at 72°C. Data collection was carried out during the extension step (30 sec at 72°C). The PCR reaction was followed by melting curve analysis to verify the specificity and identity of the resultant RT-PCR products; melting curve analysis can distinguish specific PCR products from non-specific PCR product resulting from primer-dimer formation. The primers used were 5′-GGACATGCAGTACGAGCTGA-3′ (sense), 5′-GCAGTGAAGGGCTTCTTGTC-3′ (antisense) for ALP, 5′- GTCTCACTGCCTCTCACTTG-3′ (sense), 5′-CACACATCTCCTCCCTTCTG-3′ (antisense) for Runx2, 5′-TGAGGAGGAAGTTCACTATGG-3′ (sense), 5′- TTCTTTGTGCCTGCTTTGC-3′ (antisense) for OSX, and 5′- CCCTGCCGCCCTGTACCTTGTATC-3′ (sense), 5′- AGACATTGGCTAGGGTGGCATCTGCA-3′ (antisense) for FRA1. The PCR products were heated from 65°C to 99°C at a rate of 1°C/5 sec for melting curve analysis, and the resulting data were analyzed using software provided by the manufacturer.

### Cell Fractionation

Nuclear and cytosolic extraction was performed according to the method described elsewhere [22]. Briefly, the cells were resuspended in a buffer A (20 mM Tris (pH 7.5), 1 mM EDTA, 1 mM EGTA, 1% Triton X-100, 1 µg/ml aprotinin, 1 mM phenylmethylsulfonylfluoride, and 0.5 mM sodium orthovanadate). The suspension was sonicated for 10 sec at output 4 and then centrifuged. The pellet contained the nuclear fraction. The supernatant was centrifuged at 15,000 rpm for 60 min at 4°C and contained the cytosolic fraction.

### Western Blot Analysis

Protein extracts (20 µg) were separated by 8–10% SDS-PAGE and blotted onto polyvinylidene difluoride (PVDF) membranes. Blots were washed with TBST [10 mM Tris-HCl (pH 7.6), 150 mM NaCl, 0.05% Tween-20], blocked with 5% skim milk for 1 hour, and incubated with the appropriate primary antibodies [anti-collagen type I, anti-osteopontin, and anti-β-actin (Santa Cruz Biotechnology, CA, USA) at the dilutions recommended by the supplier. The membranes were then washed, and the primary antibodies were detected with goat anti-rabbit IgG or goat anti-mouse IgG conjugated to horseradish peroxidase. Blots were developed with enhanced chemiluminescence (ECL) reagents (Santa Cruz Biotechnology) and exposed to X-ray film (Eastman-Kodak, Rochester, NY, USA).

### Immunofluorescence Staining

Cells were fixed and treated with mouse anti-collagen type I or anti-osteopontin antibody (1∶100, Santa Cruz Biotechnology, Delaware, CA) for 1 hr at room temperature. Subsequently, cells were treated with fluorescein isothiocyanate-conjugated (FITC-conjugated) anti-mouse IgG (1∶100) for 1 hr at room temperature. Fluorescence images were obtained using a fluorescence microscope (Fluoview 300, Olympus).

### Measurement of Intracellular ROS

Cellular levels of ROS were measured using 5-(and-6)-chloromethyl-2′,7′-dichlorodihydro-fluorescein diacetate (CM-H_2_DCF-DA; Molecular Probes, Eugene, OR, USA) according to the method described by Ali et al. [23]. Cells were pre-incubated with hesperetin at different concentrations for 2 hr, then 10 µM CM-H_2_DCF-DA and 30 mM glucose (HG) were added. After 40 min, DCF fluorescence was determined using a spectrofluorophotometer (RF-5301PC, Shimadzu, Japan) at an excitation wavelength of 488 nm and an emission wavelength of 530 nm.

### siRNA Transfection

Small interference RNA (siRNA) targeting β-catenin was produced using a commercial kit that contains three target sequences to β-catenin (IDT, Integrated DNA Technologies Inc., Coralville, IA, USA). The siRNA that inhibited β-catenin expression the most as determined by western blotting analysis was used in further experiments. Briefly, cells were transfected for 24 hr with either siRNA specific to β-catenin (20 nM) or negative control siRNA (scrambled), using *Trans*IT-TKO transfection reagents (Mirus, Madison, WI, USA) according to the manufacturer’s instructions, before being subjected to the various treatments.

### Statistical Analysis

All data are expressed as means ± standard deviations (S.D.). One-way ANOVA was used for multiple comparisons (Duncan’s multiple range test), using SPSS software ver. 10.0. *P* values < 0.05 were considered significant.

## Results

### High Glucose Decreases the Osteogenic Activity of PDLSCs

**Figure 1:**
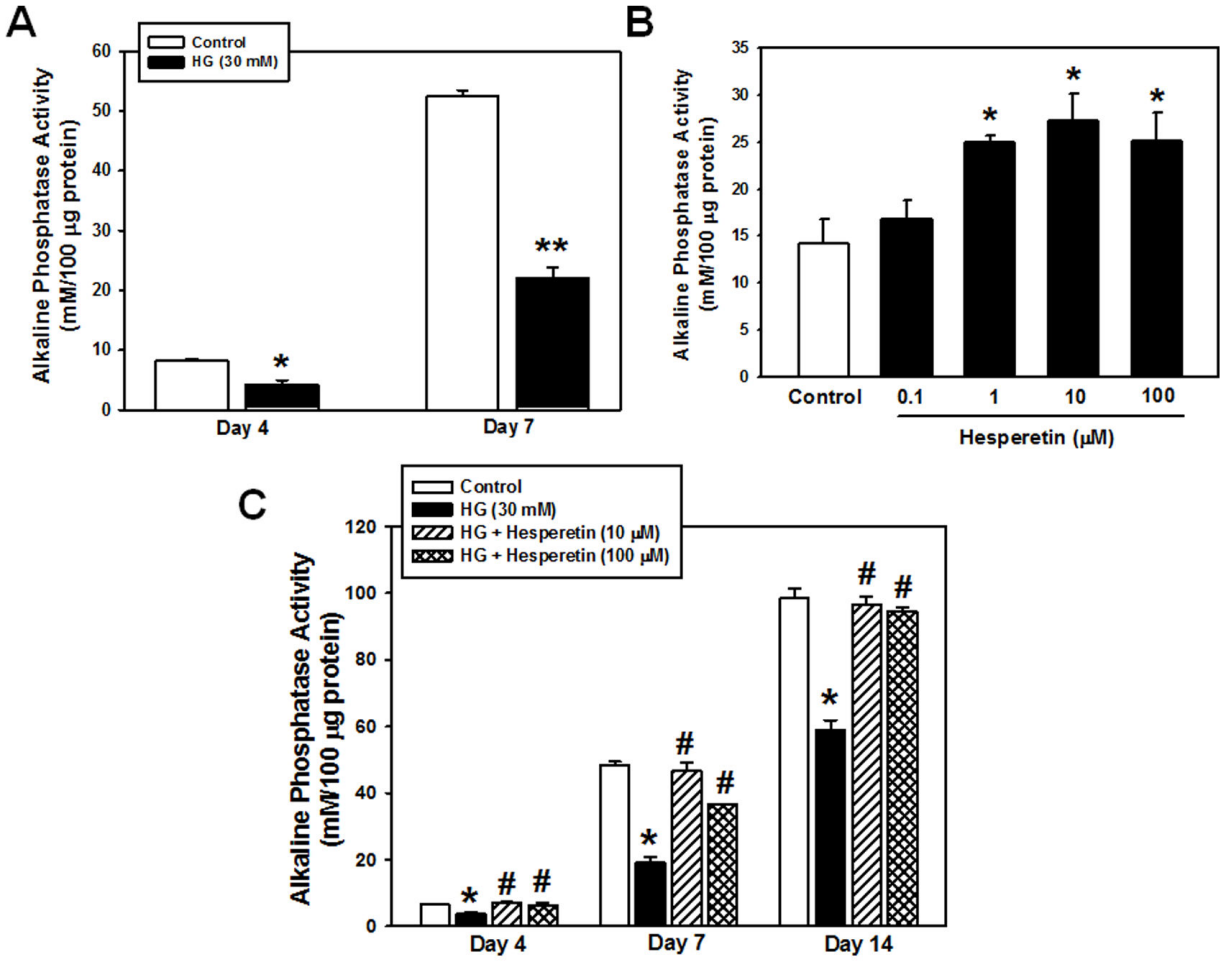
Effect of high glucose (HG) and hesperetin on osteogenic differentiation of PDLSCs. Cells were incubated in osteogenic medium with (A) 30 mM glucose (HG) or (B) hesperetin (0.1, 1, 10, 100 µM) for 4 and 7 days, respectively, and then ALP activity was assessed as described in the Materials and Methods. (C) Cells were pretreated with hesperetin at concentrations of 10 and 100 µM for 2 hr before HG exposure and ALP activity was determined after 4, 7, 14 days of osteogenic induction. The values reported are the means ± S.D. of five independent experiments. *P<0.05, **P<0.001 vs. control value, or ^#^ *P*<0.05 vs. HG treatment alone.

To examine the effect of a high level of glucose on osteogenesis of PDLSCs, cells were cultivated in medium containing NG (5.5 mM D-glucose as a control) or HG (30 mM D-glucose) reflecting physiological or diabetic blood glucose levels, respectively, then differentiated towards an osteoblastogenic lineage for 4 or 7 days. Subsequently, the ALP activity of PDLSCs was analyzed as a marker of osteogenic differentiation. Figure 1A shows that the ALP activity in cells cultured with HG was significantly lower than that of the control group at day 4 (1.7-fold decrease vs. control; *p*<0.05), which represents an early stage of osteogenic differentiation, and much more attenuated at day 7 (2.4-fold decrease vs. control; *p*<0.001).

### Effect of Hesperetin on Osteogenic Differentiation of PDLSCs

We next investigated the effect of hesperetin on osteogenic differentiation of PDLSCs under NG conditions. As shown in Fig. 1B, treatment of cells with 1 µM hesperetin resulted in a significant increase in ALP activity (1.8 fold increase vs. control; *p*<0.05). As the hesperetin concentration increased, ALP activity increased and a marked increase was observed at 10 µM hesperetin (2-fold increase vs. control; *p*<0.05).

To assess whether hesperetin counteracted the HG-induced suppression of PDLSC osteogenesis, cells were incubated with hesperetin (10 and 100 µM) prior to HG exposure. As shown in Fig. 1C, pretreatment of cells with hesperetin inhibited the HG-induced decrease in ALP activity.

### Effects of Hesperetin on Osteogenic-associated Gene Expression

**Figure 2:**
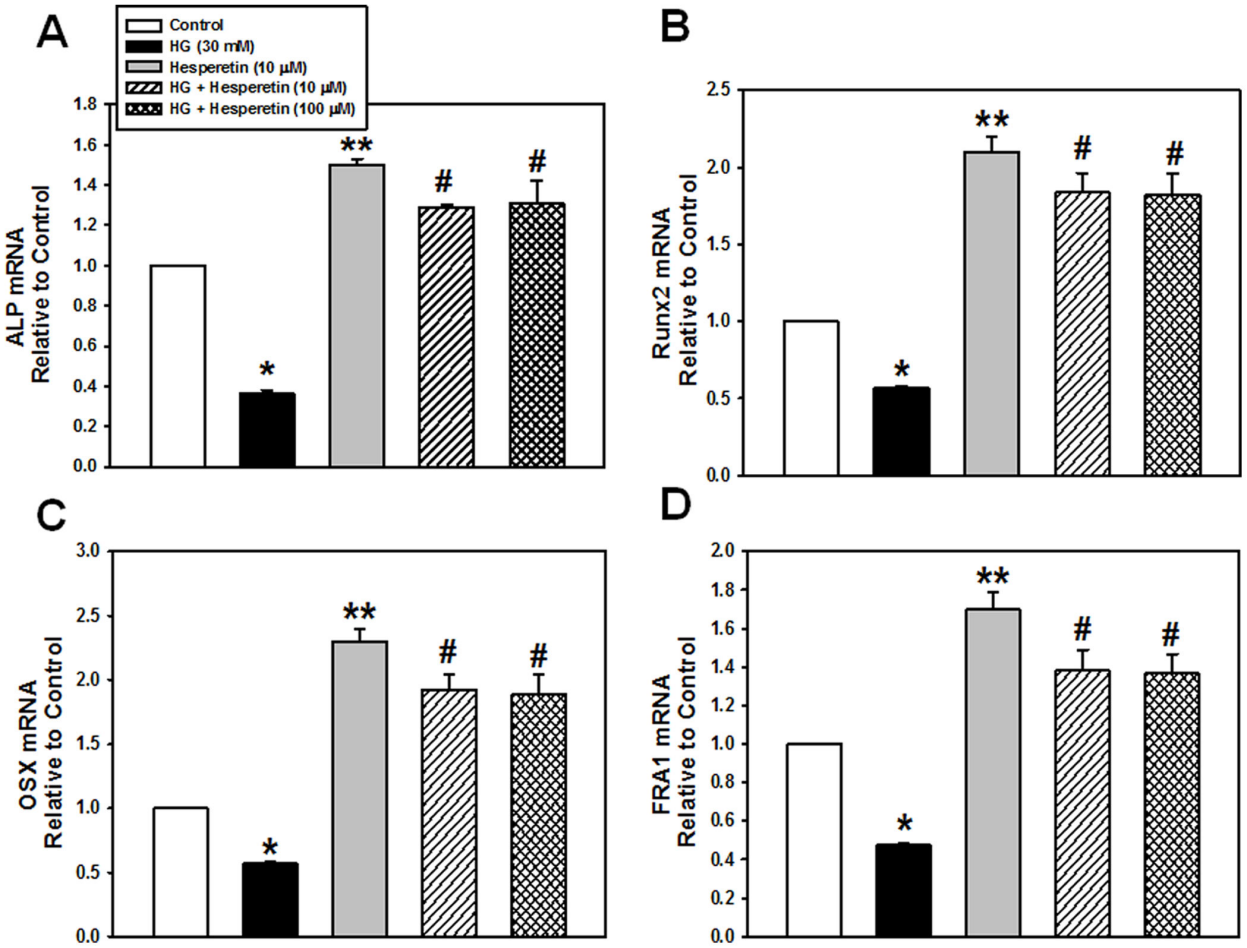
Effects of hesperetin on the high glucose-suppressed mRNA expression of ALP, Runx2, OSX, and FRA1. Cells were pretreated with hesperetin at concentrations of 10 and 100 µM for 2 hr before HG exposure and the mRNA levels of (A) ALP, (B) Runx2, (C) OSX, and (D) FRA1 were analyzed using real time RT-PCR after 4 days of osteogenic induction. The values reported are the means ± S.D. of three independent experiments. *P<0.05, **P<0.001 vs. control values, or ^#^ *P*<0.05 vs. HG treatment alone.

To further investigate the effects of hesperetin on PDLSCs exposed to HG, we determined the mRNA expression levels of the following osteogenic target genes: ALP, Runx2, OSX, and FRA1, using real time RT-PCR. Cells incubated with HG expressed lower levels of these marker genes than control cultures. However, treatment with hesperetin alone markedly increased the mRNA expression of osteogenic marker genes and pretreatment with hesperetin recovered them under HG conditions (Fig. 2).

### Effects of Hesperetin on Protein Levels of Osteogenic Factors

**Figure 3:**
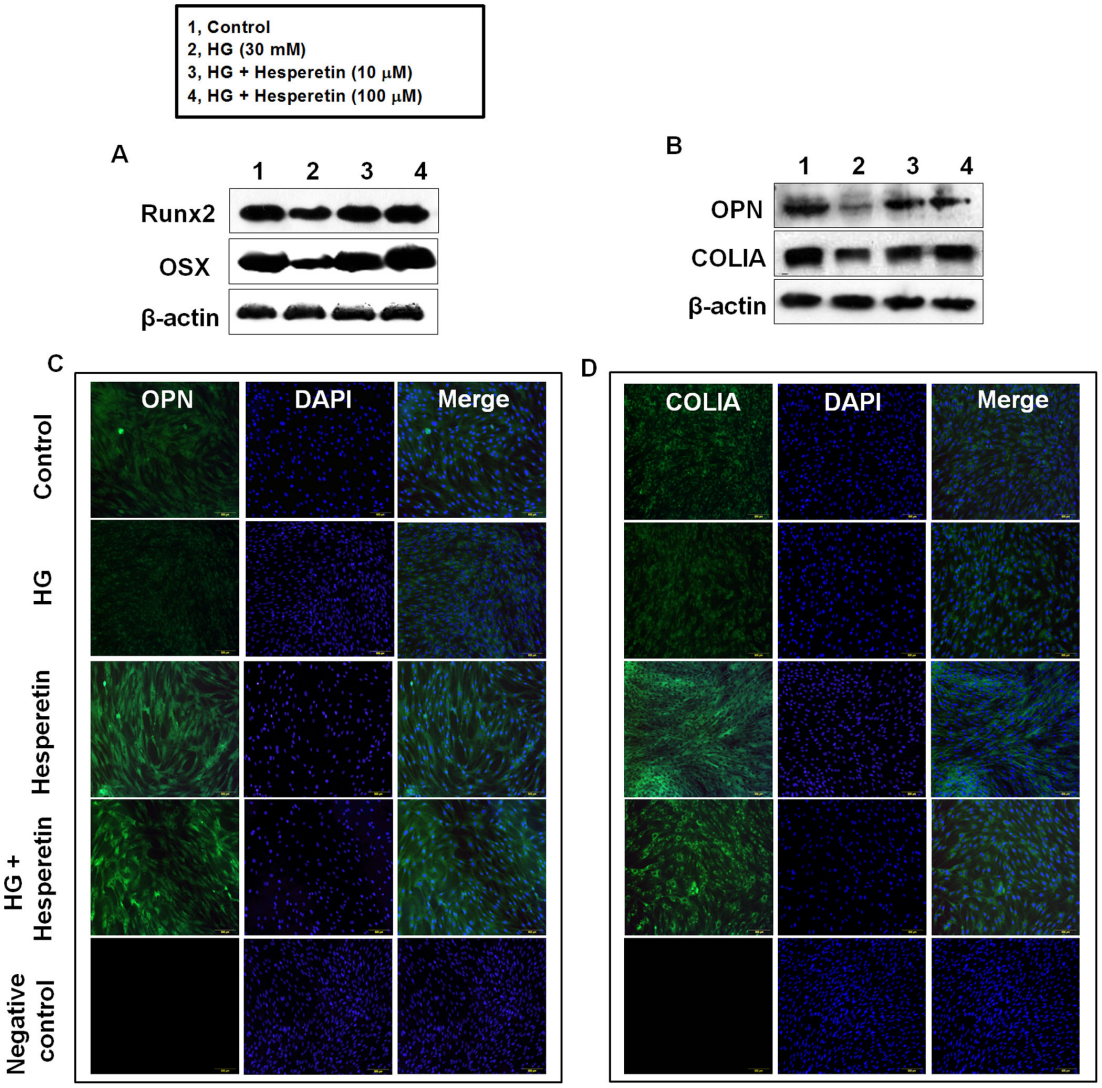
Effects of hesperetin on the high glucose-mediated suppression of Runx2, OSX, OPN, and COLIA protein levels. (A) Cells were pretreated with hesperetin at concentrations of 10 and 100 µM for 2 hr before HG incubation and then protein levels of (A) Runx2, OSX, (B) OPN, and COLIA were determined by western blot analysis using total protein lysates. Cells were incubated with hesperetin in the presence of HG for 4 days then (C) OPN and (D) COLIA were detected by immunostaining. Negative control staining using secondary antibody alone to assess nonspecific fluorescence. Nuclei were stained with DAPI (blue staining). A representative result from four independent experiments is shown.

We also analyzed the effects of hesperetin on the osteogenic differentiation of PDLSCs by following the protein expression of Runx2, OSX, OPN, and COLIA, which are osteogenic markers, four days after osteogenic induction. Western blot analysis showed that the level of each protein decreased in cells incubated with HG relative to control cells. When cells were pretreated with 10 or 100 µM hesperetin prior to HG exposure, expression of these proteins was higher than that observed in HG cultures (Fig. 3A, B). Moreover, immunofluorescence staining for OPN and COLIA confirmed that hesperetin treatment enhanced the differentiation of PDLSCs into am osteogenic lineage (Fig. 3C, D).

### Involvement of ROS and PI3K/Akt Signaling in Hesperetin-induced Osteogenic Differentiation

**Figure 4:**
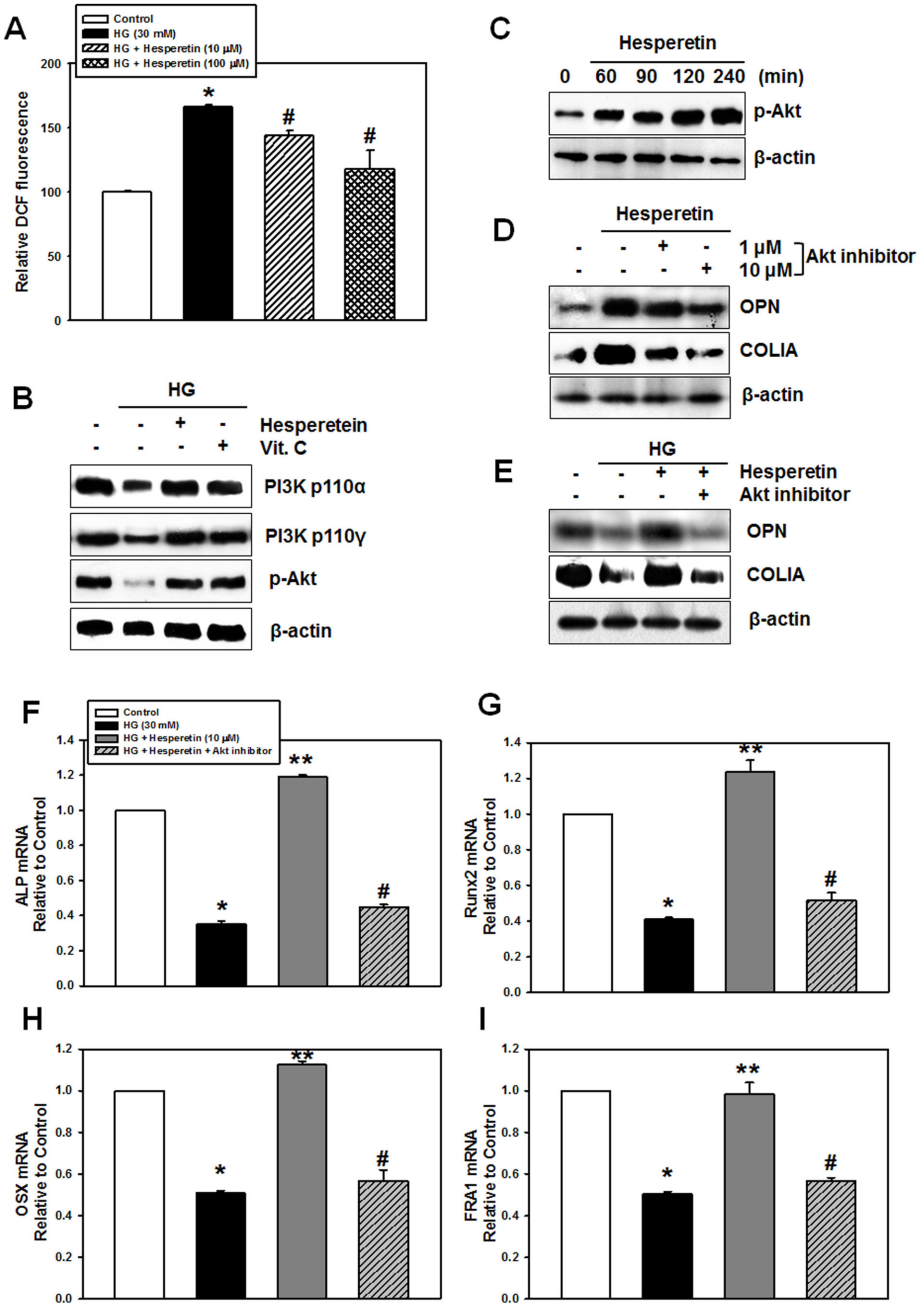
Effect of hesperetin on intracellular ROS levels and PI3K/Akt signaling. (A) Cells were preincubated with hesperetin at different concentrations for 2 hr then 10 µM CM-H_2_DCF-DA and high glucose were added. After 40 min, DCF fluorescence was determined using a spectrofluorophotometer. (B) Protein levels of PI3K p110α and γ isoforms as well as p-Akt were determined after cells were incubated with hesperetin or vitamin C in the presence of HG for 4 days. (C) Phosphorylation of Akt by hesperetin (10 µM) was assessed with cells of 7 day-osteogenic induction. (D, E) OPN and COLIA levels were determined after incubation of cells with Akt inhibitor in the presence of HG, hesperetin, or HG+hesperetin. (F–I) The mRNA levels of osteogenic target genes were determined after incubation with Akt inhibitor in the presence of HG or HG+hesperetin. The values reported are the mean ± S.D. of three independent experiments. *P<0.05 vs. control values, **P<0.05 vs. HG treatment alone, or ^#^ *P*<0.05 vs. HG+hesperetin.

Hesperetin is known to have antioxidant and free radical scavenging ability [24], [25]. When cells were incubated with HG, intracellular ROS level was increased up to 66% compared to control cells. However, pretreatment of cells with hesperetin inhibited the HG-induced ROS increase, suggesting that the scavenging activity of hesperetin is involved in PDLSC osteogenesis (Fig. 4A). Next, we examined the role of PI3K/Akt signaling in hesperetin-induced osteogenic differentiation. As shown in Fig. 4B, a high glucose level significantly decreased the protein expression of PI3K p110α and γ isoforms as well as Akt phosphorylation compared to the control cultures. However, pretreatment of cells with hesperetin or vitamin C increased expression of these proteins under HG conditions. We also examined the effect of hesperetin on the phosphorylation of Akt under NG conditions; a significant increase in the level of p-Akt (ser 473) was first observed at 60 min and maintained up to 240 min (Fig. 4C). To examine the association between PI3K/Akt signaling and hesperetin-induced osteogenesis of PDLSCs, cells were pretreated with 1 or 10 µM of Akt inhibitor before hesperetin application. As shown in Fig. 4D, the hesperetin-induced increase in the protein levels of OPN and COLIA was significantly attenuated by addition of Akt inhibitor, but inhibitor alone did not change these protein levels compared to control cells (Figure S2). Moreover, under HG conditions, the HG-mediated decrease in the protein levels of OPN and COLIA as well as the mRNA expression of osteogenic marker genes were attenuated by hesperetin, but this did not occur in the presence of Akt inhibitor, suggesting that hesperetin promotes PDLSC osteogenesis under HG conditions by activating the PI3K/Akt pathway (Fig. 4E–I).

### Hesperetin Stimulates the Canonical Wnt/β-catenin Signaling Pathway

**Figure 5:**
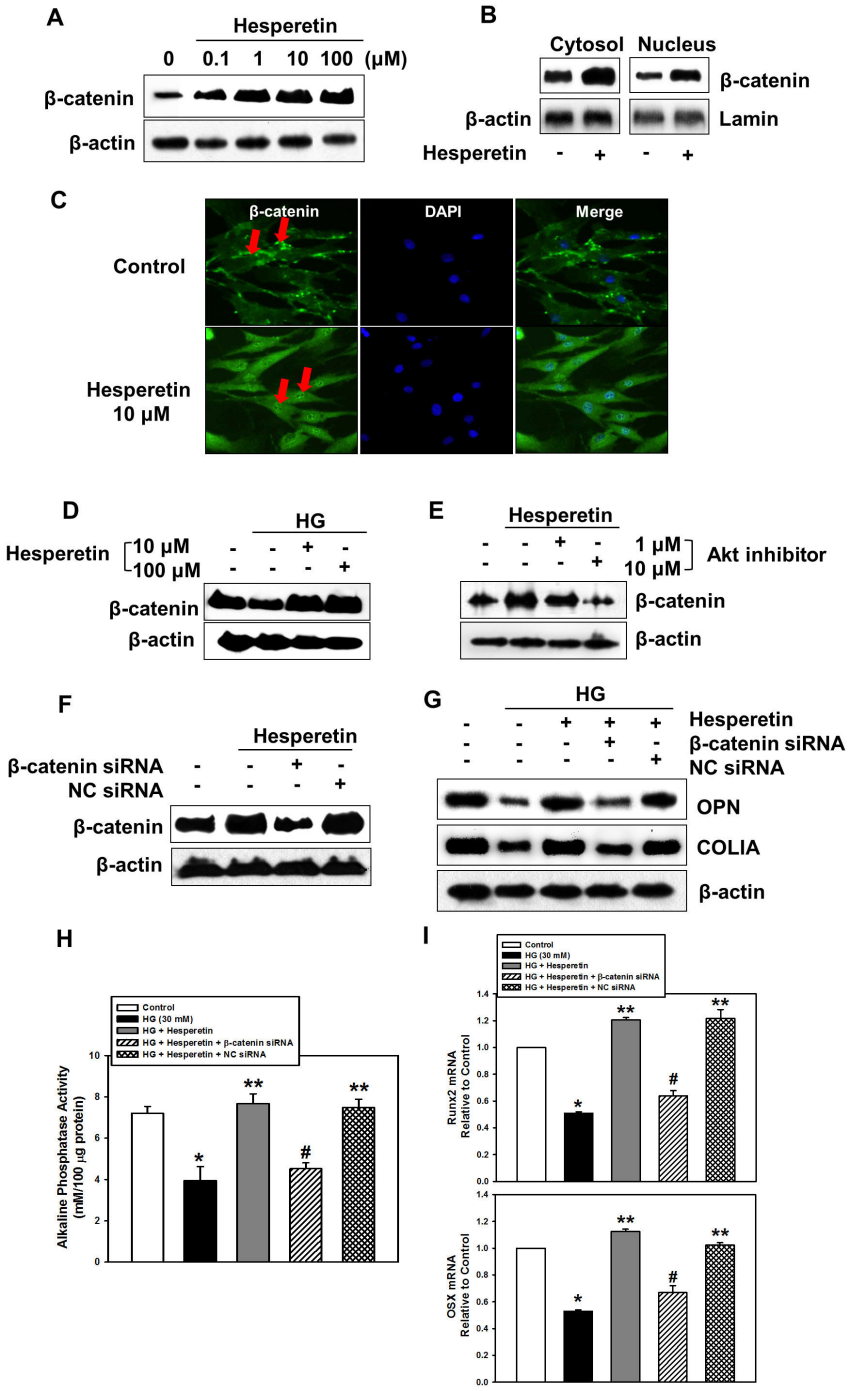
Effect of hesperetin on Wnt/β-catenin signaling. (A) Protein levels of β-catenin and (B, C) nuclear translocation of β-catenin were assessed by Western blot analysis or immunofluorescence staining after cells were incubated with hesperetin. Protein levels of β-catenin were determined after cells were conditioned with (D) hesperetin in the presence of HG and (E) hesperetin+Akt inhibitor. (F) Changes in β-catenin levels were determined according to transfection. (G) Protein levels of OPN and COLIA, (H) ALP activity, and (I) the mRNA levels of Runx2 and OSX were measured after cells were transfected with β-catenin siRNA for 24 hr and further incubated with hesperetin and HG for 24 hr. The values reported are the mean ± S.D. of three independent experiments. *P<0.05 vs. control values, **P<0.05 vs. HG treatment alone, or ^#^ *P*<0.05 vs. HG+hesperetin.

We further evaluated the role of Wnt/β-catenin signaling in hesperetin-induced osteogenesis of PDLSCs. Figure 5A shows that hesperetin increased protein expression of total β-catenin dose-dependently under NG conditions. In order to validate that hesperetin indeed stimulates β-catenin translocation, cells were exposed to hesperetin then cytosol and nuclear proteins were isolated. Western blot analysis demonstrated that hesperetin increased the protein levels of β-catenin in the cytosol and nucleus (Fig. 5B). Subsequently, we confirmed that hesperetin induced nuclear translocation of β-catenin using immunofluorescence staining (Fig. 5C). In contrast, cultures incubated with HG had lower β-catenin expression than control cultures. However, addition of 10 or 100 µM hesperetin attenuated the HG-induced decrease in expression of β-catenin (Fig. 5D). To elucidate the relationship between PI3K/Akt and Wnt/β-catenin signaling in the process of osteogenesis, cells were pre-incubated with Akt inhibitor before hesperetin treatment. As shown in Fig. 5E, the hesperetin-induced increase in β-catenin levels was attenuated by the Akt inhibitor. However, knock-down of β-catenin did not affect the phosphorylation of Akt by hesperetin, suggesting that PI3K/Akt signaling acts as an upstream signaling molecule for Wnt/β-catenin pathways (Figure S3).

Finally, we determined the effect of Wnt/β-catenin signaling on the regulation of osteogenic factors. To confirm the efficacy of the β-catenin-specific siRNA, cells were transfected with either β-catenin or negative control siRNA using *Trans*IT-TKO transfection reagents. Twenty-four hours after transfection, cells were incubated with hesperetin for 24 hr. Figure 5F shows that the hesperetin-induced increase in levels of β-catenin was reduced markedly by siRNA transfection of siRNA specific to β-catenin, where negative control siRNA transfection did not affect β-catenin protein levels. Under HG conditions, β-catenin ablation by siRNA transfection diminished the hesperetin-induced increase in OPN and COLIA protein expression (Fig. 5G). Moreover, the patterns of ALP activity and the mRNA expression of osteogenic marker genes were consistent with Western blot analysis of OPN and COLIA suggesting that the canonical Wnt/β-catenin signaling is involved in hesperetin-induced osteogenesis in PDLSCs (Fig. 5H, I).

## Discussion

In the present study, we demonstrated the inhibitory effect of a diabetic glucose level on osteogenic differentiation of PDLSCs, and showed how hesperetin could stimulate the osteogenesis of PDLSCs in this high glucose condition. A high concentration of mannitol was used as an osmotic control, and the corresponding values were similar to those in NG cultures (data not shown). High blood glucose is the prominent pathogenic factor that leads to diabetic complications including bone disease and oral health disorders. Increasing evidence suggests that high glucose concentrations inhibit osteoblast proliferation and osteogenic factor secretion [26], [27], because high glucose levels can directly impair osteoblastic functions. A previous study showed that PDL cells from insulin-dependent diabetic patients had altered functions; they formed mineralized tissue and responded to exogenous growth factors [28]. Moreover, an in vitro experiment demonstrated that high glucose inhibited the proliferation and mineralization of PDL cells [29]. However, the osteoblastic differentiation of PDLSCs under high glucose conditions has not been thoroughly explored. In the present study, we demonstrated that down-regulation of osteogenesis in PDLSCs cultured in high glucose may explain the delay in periodontal regeneration and healing observed in diabetic patients.

In the present study, hesperetin, similar to other polyphenols with osteogenic effects [30], [31], stimulated the osteogenic differentiation of PDLSCs. It has previously been demonstrated that hesperetin can influence osteoblast differentiation via the bone morphogenic protein (BMP) signaling pathway [32]. Moreover, treatment of osteoblasts with physiological concentrations of hesperetin significantly augmented the mRNA expression of ALP, Runx2, and osterix [32]. Despite a different cell model, when osteoblasts were treated with hesperetin under diabetic glucose conditions, ALP activity and collagen content increased markedly [18]. Similarly, we demonstrated in this study that hesperetin prevented high glucose-mediated PDLSC dysfunction resulting in defective osteogenic differentiation. Based on these results, hesperetin is a promising potential candidate for bone regeneration.

Long-term exposure to high glucose concentrations induces oxidative stress, which adversely affects cellular functions. Thus, removal of oxidized factors by antioxidants is part of the cellular defense system against oxidative stress [33]. In the present study, we examined the effect of hesperetin on high glucose-induced ROS over-production in PDLSC cultures. As expected, high glucose exposure markedly increased ROS generation, which was reversed by pretreatment of cells with 10 and 100 µM hesperetin. These results are consistent with those of a previous study that showed that hesperetin induced a decrease in ROS in mouse embryonic stem cells treated with hydrogen peroxide [34]. These findings indicate that hesperetin, due to its radical scavenging ability, functions in cellular defense. Previous studies have shown that oxidative stress suppresses osteogenic differentiation in various cellular systems [35]–[37]. Indeed, regulatory transcription networks between redox-responsive elements and the OPN gene have been reported [38]. In this respect, the antioxidant activity of hesperetin could be linked to its osteogenic capability, suggesting that use of this compound may be a novel strategy to prevent the inhibitory effect of oxidative stress on osteogenesis of PDLSCs in a diabetic state.

Cell signaling pathways involved in osteogenesis can be activated by the interactions of a compound with extracellular or intracellular molecules. Among them, the PI3K/Akt and Wnt/β-catenin pathways have been the focus of recent work, and both have emerged as critical for bone development and skeletal remodeling [39], [40]. Recent genetic studies of PI3K signaling revealed that Akt and its downstream targets are critical regulators of bone formation and osteoblastic survival [41], [42]. In the present study, hesperetin increased protein levels of PI3K isoforms and the phosphorylation of Akt, which is consistent with a previous study that demonstrated that hesperetin induced PI3K activation in PC12 cells [43]. Our results suggest a significant synergistic effect between the PI3K/Akt and Wnt/β-catenin pathways. Hesperetin-stimulated Wnt/β-catenin signaling was associated with PI3K/Akt activation. In contrast, inhibition of Akt or Wnt/β-catenin signaling blocked the preventive effect of hesperetin against high glucose-suppressed osteogenesis of PDLSCs, suggesting that hesperetin increases the osteogenic capacity of PDLSCs by activating the PI3K/Akt and Wnt/β-catenin signaling pathways.

**Figure 6:**
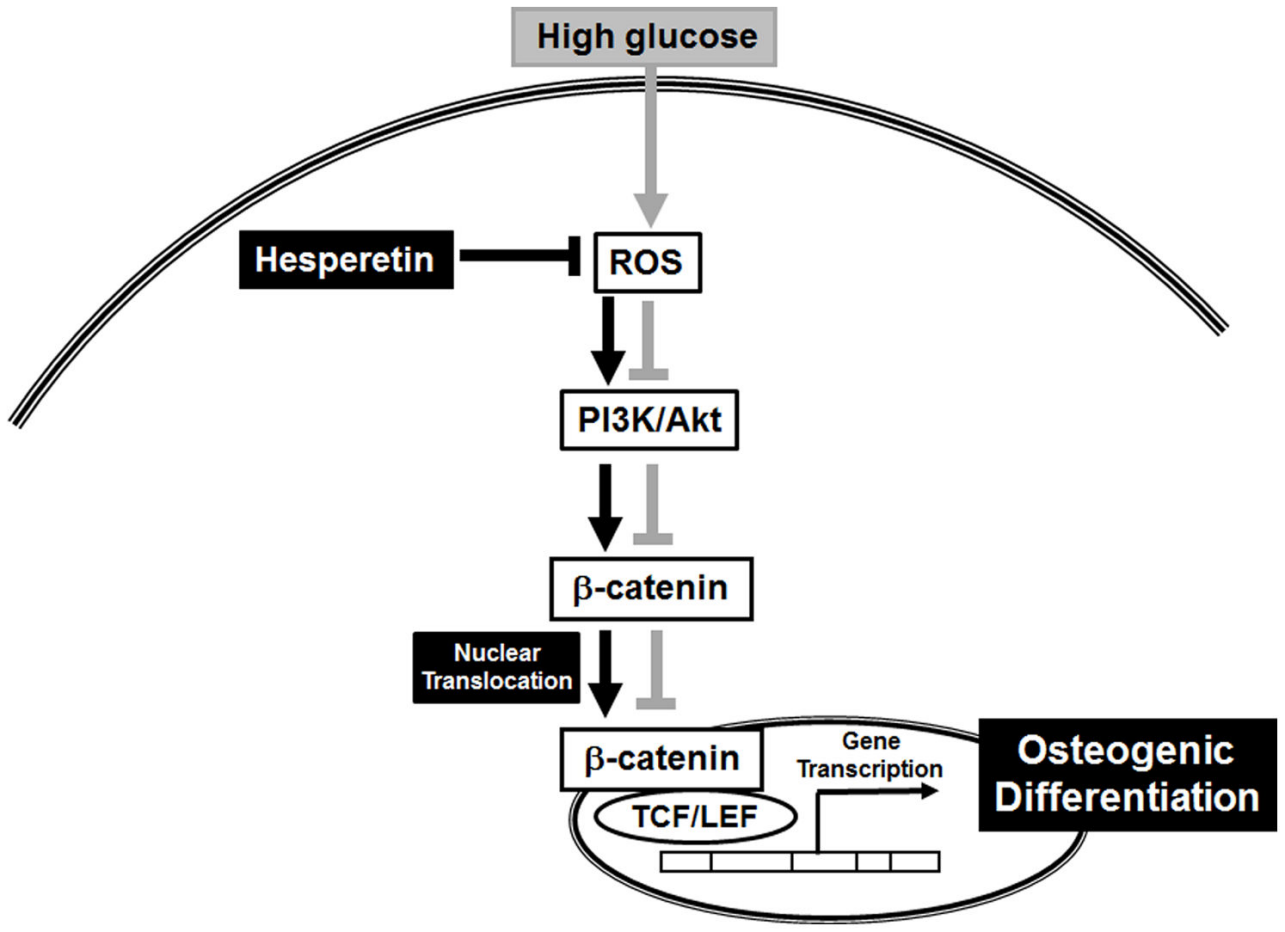
Hypothesized model of the signaling pathways underlying the rescuing effects of hesperetin on high glucose-exposed PDLSCs. High glucose increases ROS generation, which inhibits PI3K/Akt signaling and Wnt/β-catenin. Hesperetin suppresses ROS production and activates PI3K/Akt signaling and Wnt/β-catenin to induce the translocation of β-catenin into the nucleus, which leads to the osteogenic differentiation of PDLSCs. In this scheme, grey lines are proposed pathways affected by high glucose concentrations and black lines are hesperetin-stimulated pathways.

In conclusion, we demonstrated that hesperetin is a promising bioactive compound that preserves the osteogenic capacity of PDLSCs under diabetic conditions. Our findings suggest that hesperetin alleviates the inhibitory effect of high glucose on the osteoblastic differentiation of PDLSCs through ROS scavenging and modulation of PI3K/Akt and Wnt/β-catenin signaling (Fig. 6). Hesperetin is promising biochemical candidate to control PDLSC behavior and can potentially play a regenerative role in restoring the function of PDLSCs in diabetes-induced periodontal diseases.

## Supporting Information

Figure S1 Colony-forming assay. To assess the colony-forming efficiency, cells at passage 1 were seeded onto a 35 mm dishes. After 5 days of culture, cultures were fixed with 4% formalin, and then stained with 0.5% crystal violet (Sigma–Aldrich Co.). A representative result from four independent experiments is shown.
(TIF)

Figure S2 Protein levels of OPN and COLIA were determined after incubation of cells with Akt inhibitor in the presence or absence of hesperetin. A representative result from three independent experiments is shown.
(TIF)

Figure S3 Phosphorylation of Akt was measured after cells were transfected with β-catenin siRNA for 24 hr and further incubated with hesperetin and HG for 24 hr. A representative result from three independent experiments is shown.
(TIF)
