# Supplementary figures and images for: Hesperetin Alleviates the Inhibitory Effects of High Glucose on the Osteoblastic Differentiation of Periodontal Ligament Stem Cells
Source: PLoS One. 2013 Jun 28;8(6):e67504. doi: 10.1371/journal.pone.0067504 (PMC3696082; doi:10.1371/journal.pone.0067504)

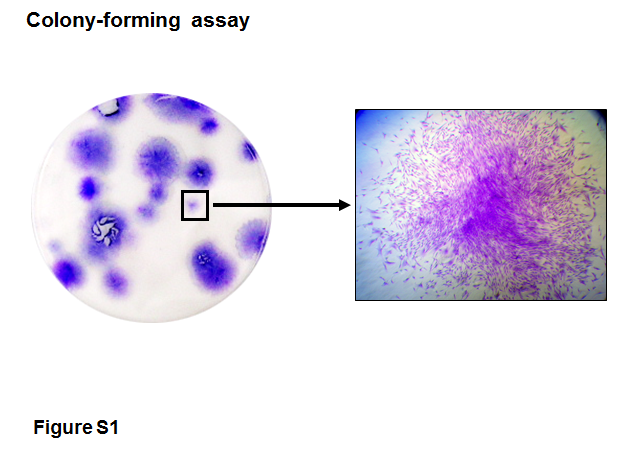

Supplement: Figure S1 — Colony-forming assay. To assess the colony-forming efficiency, cells at passage 1 were seeded onto a 35 mm dishes. After 5 days of culture, cultures were fixed with 4% formalin, and then stained with 0.5% crystal violet (Sigma–Aldrich Co.). A representative result from four independent experiments is shown. (TIF) [file pone.0067504.s001.tif]

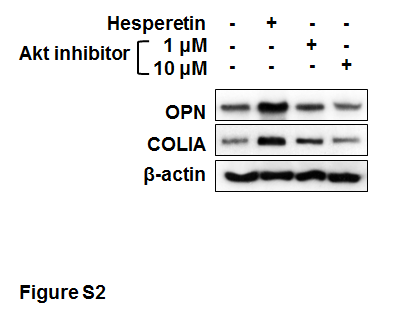

Supplement: Figure S2 — Protein levels of OPN and COLIA were determined after incubation of cells with Akt inhibitor in the presence or absence of hesperetin. A representative result from three independent experiments is shown. (TIF) [file pone.0067504.s002.tif]

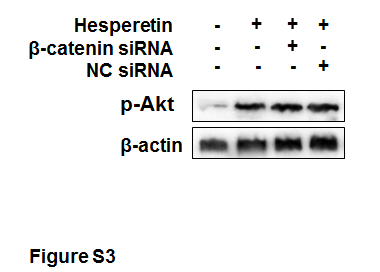

Supplement: Figure S3 — Phosphorylation of Akt was measured after cells were transfected with β-catenin siRNA for 24 hr and further incubated with hesperetin and HG for 24 hr. A representative result from three independent experiments is shown. (TIF) [file pone.0067504.s003.tif]
